# On the Edge

**DOI:** 10.4269/ajtmh.24-0459

**Published:** 2024-10-01

**Authors:** Madhusudan Samprathi

**Affiliations:** Department of Pediatrics, All India Institute of Medical Sciences, Bibinagar, India

There was an unusual air of mirth and palpable excitement as I entered the pediatric intensive care unit (PICU) to start the evening rounds. A young girl was engaged in an animated conversation with the nurses. As I entered, the nurses rose to join me while the girl, Jyoti,^†^ moved away. Once we moved to the second bed, I realized that Jyoti was the elder sister of an infant boy admitted with pneumonia. The mother at the bedside narrated the story of his illness to me. He had fevers and a cough for a few days. Then he started breathing fast. Eventually he became lethargic. The disheveled appearance of the mother and her two children made me curious about their background. I came to know that she begged for a living at the bus station nearby. Her husband had abandoned her and the children a few months back. An older unrelated woman, also a beggar, had offered shelter to them. It appeared that there were more such women and their children in that locality who lived together supporting each other.

The nurse told me that although they were admitted in the afternoon, they had not had a single meal yet that day. After admission, another family whose child was admitted with us had offered Jyoti and her mother a meal. Although Jyoti had initially appeared dull and tired, she had quickly gobbled up what she could and transformed into a chatterbox, engaging the entire PICU team in her lively banter. Meanwhile, our patient here, the 2-month-old boy, was visibly tachypneic and on high-flow nasal oxygen. We decided to help the family in whatever way we could, in addition to caring for the young boy. I was relieved that as long as they were in the hospital, the mother-daughter duo would be getting three square meals a day.

When I came in the evening to check on the boy, Jyoti seemed to be busy chatting with parents of other admitted children. She was excited by the lights and beeps of the monitors and curious about the myriad equipment around her. She bombarded the nurses with questions on all that she saw around her. The nurses found her an engaging companion, as she amused them with incidents from her life. When her mother was away, she was the sole caretaker of her young brother. She could be seen animatedly speaking to her brother, and he responded with coos and squeals, amidst his fast breaths and the prongs in his tiny nose! On one occasion when the mother was away for too long, we panicked and almost informed the authorities that she had absconded. Jyoti, however, remained as cool as ever, shrugging it off saying “she must have gone to get food for us!”

Fortunately, the boy responded in 48 hours and could be shifted out of the ICU. In another day, he was discharged. Although we had given the mother strict instructions to follow up with us, I was not surprised when she did not. She must have been preoccupied with achieving her biggest goal everyday—feeding her two small children and herself.

Occasionally, at times when busy with work and ravenous, I remember Jyoti and her family and many more like them, who may not get a single, proper meal a day. When as an adult I cannot tolerate hunger, I cringe at the thought that there are so many children around the world who have to sleep hungry. Their biggest battle in life is so basic. Yet, living on the edge does not dent their energy and enthusiasm. The “haves” seem to sulk through their battles, whereas the “have-nots” smile through theirs, silently imparting extraordinary life lessons from their ordinary lives.

## Footnotes

^†^
Pseudonym: “Jyoti” in Sanskrit means “light” and symbolizes knowledge, learning, and enlightenment.

